# Role of Modified Atmosphere in Pest Control and Mechanism of Its Effect on Insects

**DOI:** 10.3389/fphys.2019.00206

**Published:** 2019-03-12

**Authors:** Yu Cao, Kangkang Xu, Xiaoye Zhu, Yu Bai, Wenjia Yang, Can Li

**Affiliations:** Guizhou Provincial Key Laboratory for Rare Animal and Economic Insect of the Mountainous Region, Department of Biology and Engineering of Environment, Guiyang University, Guiyang, China

**Keywords:** modified atmosphere, physiological adaptation, pest control, hypoxia, molecular mechanisms

## Abstract

Pests not only attack field crops during the growing season, but also damage grains and other food products stored in granaries. Modified or controlled atmospheres (MAs or CAs) with higher or lower concentrations of atmospheric gases, mainly oxygen (O_2_), carbon dioxide (CO_2_), ozone (O_3_), and nitric oxide (NO), provide a cost-effective method to kill target pests and protect stored products. In this review, the most recent discoveries in the field of MAs are discussed, with a focus on pest control as well as current MA technologies. Although MAs have been used for more than 30 years in pest control and play a role in storage pest management, the specific mechanisms by which insects are affected by and adapt to low O_2_ (hypoxia) and high carbon CO_2_ (hypercapnia) are not completely understood. Insect tolerance to hypoxia/anoxia and hypercapnia involves a decrease in aerobic metabolism, including decreased NADPH enzyme activity, and subsequently, decreases in glutathione production and catalase, superoxide dismutase, glutathione-S-transferase, and glutathione peroxidase activities, as well as increases in carboxyl esterase and phosphatase activities. In addition, hypoxia induces energy and nutrient production, and in adapted insects, glycolysis and pyruvate carboxylase fluxes are downregulated, accompanied with O_2_ consumption and acetate production. Consequently, genes encoding various signal transduction pathway components, including epidermal growth factor, insulin, Notch, and Toll/Imd signaling, are downregulated. We review the changes in insect energy and nutrient sources, metabolic enzymes, and molecular pathways in response to modified O_2_, CO_2_, NO, and O_3_ concentrations, as well as the role of MAs in pest control. This knowledge will be useful for applying MAs in combination with temperature control for pest control in stored food products.

## Introduction

Herbivorous insects not only attack field crops during the growing season, but also damage grains stored in granaries (Weaver and Petroff, 2005; Sadeghi et al., 2011). Losses of 5–10%, and up to 40% in developing countries, caused by insects in stored products have been reported worldwide (Shaaya et al., 1997; Weaver and Petroff, 2005). Fumigation is an optimal management practice to control all stages and kinds of pests in grain bins, warehouses, and other mass grain-storage structures. Therefore, fumigation with chemical insecticides presently is the most effective and widely used method to control storage pest infestations. However, the excessive use of chemical can lead to pesticide residues in treated grain or grain products that cause human health and environmental problems as well as potential resistance development in insects (Cheng et al., 2012). Modified or controlled atmospheres (MAs or CAs) depleted in oxygen (O_2_) and/or with elevated levels of carbon dioxide (CO_2_) or other gases provide an environmentally friendly and cost-effective approach to protecting grains and other stored food products (Cheng et al., 2012).

The concentrations of atmospheric carbon dioxide (CO_2_) are rising at an accelerated rate, which greatly affects the behavior and adaptation of herbivorous insects (Guo et al., 2014). In northern latitudes, natural insect enemies might benefit from the increasing temperature for their development, which in turn might facilitate integrated pest management (Castex et al., 2018). Our focus is not on the effects of global changes in CO_2_ concentrations on insect pests, as, importantly, insects can relatively easily adapt to CO_2_, O_2_, nitric oxide (NO), or ozone (O_3_) stress by changing their physiology and thus increase their survival rate under CA or other stresses. Therefore, we focus on the potential role of CAs in pest control.

Farmers and warehouse managers are interested in using hermetic storage for pest control in stored products (Njoroge et al., 2016, 2017). Several studies have evaluated the timing of insect die-off under CAs with reduced O_2_ or increased CO_2_ (Soderstrom et al., 1990; Ofuya and Reichmuth, 2002; Gunasekaran and Rajendran, 2005). For example, exposure for 17 days to a mixture of 40% CO_2_ and 2% O_2_ resulted in 100% mortality of grain weevils, *Calandra granaria* Linnaeus (Bailey, 1955). Under gradual reduction of O_2_ to 0% in 6–9 days in hermetic conditions, maize weevils (*Sitophilus zeamais* Linnaeus) produced a significantly lower number of offspring than weevils in non-hermetic conditions (Moreno-Martinez et al., 2000). *Cadra cautella* Walker and *Tribolium castaneum* Herbst showed significantly different susceptibilities to a high CO_2_ concentration of 99.9% at different developmental stages (Husain et al., 2017).

Insects have an effective respiratory system that allows direct air inflow from the atmosphere through muscular valves called spiracles. Insects accomplish respiratory gas exchange by controlling the opening and closing of these spiracles, and ventilate their tracheal system through muscular contractions (Matthews and White, 2011). As gaseous fumigants are mainly absorbed through the respiratory system, factors that influence respiration in insects also affect fumigant uptake (Lu et al., 2009). Changes in the concentrations of O_2_, CO_2_, and other gases can potentially affect the respiration rate and hence, the rate and biochemistry of metabolization and incorporation, and ultimately, the toxicity of a fumigant (Lu et al., 2009).

An MA with depleted O_2_ (hypoxia) and/or elevated CO_2_ (hypercapnia) is an environmentally friendly alternative to fumigants, which are currently widely used for stored-grain pest control (Cheng et al., 2012; Li et al., 2012; Mehmood et al., 2018). Although MAs have been used as a safe alternative to conventional fumigants for more than 30 years, the specific mechanisms by which insects are affected by and adapt to hypoxia and hypercapnia remain poorly understood (Boyer et al., 2012; Ingabire et al., 2013). Certain gas compositions, e.g., 100% CO_2_, 75% CO_2_, and 25% N_2_, and 22 ppm O_3_, can be used together with temperature control to effectively control pests in stored grains (Husain et al., 2015). MA treatments using CO_2_, O_2_, N_2_, and/or O_3_ together with other measures, e.g., controlled temperature or humidity, provide important means to reduce insect survival or postharvest disinfestation (Boardman et al., 2011). MA treatments usually involve either low O_2_ (0–11.5 kPa) or high CO_2_ (18–90 kPa) and are applied with augmented-temperature sterilization to combat pests in stored products.

## Modified Atmosphere Gases Commonly Used for Pest Control and their Toxicities

Ambient atmosphere consists of approximately 79% N_2_, 20–21% O_2_, and 0.04% CO_2_. MAs with hypoxia and/or hypercapnia in airtight storage, with O_2_ maintained at a level sufficient for insect development, have been used for preventing insect damage in stored grains (Banks and Annis, 1990; Fleurat-Lessard, 1990; Riudavets et al., 2009; Sanon et al., 2011; Navarro et al., 2012; Rasool et al., 2017).

MAs generally involve O_2_, CO_2_, NO, and O_3_. Insect tolerance to hypoxia and hypercapnia critically affects insect control (Cui et al., 2017). O_2_ is critical for the survival of aerobic life. However, oxidative injury can be induced by a too low or too high (hyperoxia) O_2_ level in organisms, which will induce morbidity and mortality (Zhao and Haddad, 2011). For example, egg laying in insects decreases with increasing CO_2_ concentration (Azzam et al., 2010). CO_2_ toxicity increases in a concentration-dependent manner, as reported for *Stegobium paniceum* Linnaeus and *Oryzaephilus surinamensis* Linnaeus (Cao et al., 2015a,b). Adult insects and larvae show different susceptibilities to CO_2_ stress. For example, at 90% CO_2_, the LT_50_ and LT_99_ of adult insects reportedly are 6.89 and 15.83 h, and those of larvae 18.76 and 60.58 h, respectively (Cao et al., 2015a). A 12-hour exposure to 80% CO_2_ and 20% N_2_ at 32.2°C resulted in 100% mortality of pupae of *Plodia interpunctella* Hübener (Sauer and Shelton, 2002). Larval mortality in *Ephestia cautella* Walker (Husain et al., 2015) and mosquito (Garcia et al., 2014) was higher after 48-h than after 24-h exposure to 100% CO_2_ or 75% CO_2_ at 25°C. An MA with 8% O_2_, 60% CO_2_, and 32% N_2_ at 30°C killed 100% of 4th instar larvae of *E. cautella* within 72 h, and resulted in 95% mortality in *Amyelois transitella* Walker after 60-h exposure at 27°C (Brandle et al., 1983). Under the same MA, the mortality of *E. cautella* significantly increased when the temperature was increased from 25 to 35°C (Husain et al., 2015). These results indicate that an MA combined with higher temperature is an effective method for pest control in stored products in future.

NO is a potent fumigant that shows excellent control effect on all insects, regardless of their life stage (Liu, 2013, 2015, 2016; Liu and Yang, 2016; Yang and Liu, 2018). However, the application of NO MAs should follow a logical order (Li et al., 2009; Riudavets et al., 2009; Navarro, 2012). For example, when NO is used with nitrogen (N_2_) in an airtight fumigation chamber to protect fresh fruit and vegetables against pests infection, N_2_ should be flushed into the chamber first, to create an ultralow oxygen (ULO) environment, followed by injection of NO (Liu et al., 2016, 2017). Because nitrogen dioxide (NO_2_) will be produced when NO reacts with O_2_, NO fumigation must be applied under ULO conditions and under low temperature (Liu, 2013).

As a natural atmosphere component, O_3_ can rapidly decompose to molecular oxygen, without leaving residues (Lu et al., 2009). Gaseous O_3_ is used in food processing (Palou et al., 2002; Forney et al., 2007; Wei et al., 2007), and as a fumigant against stored-product pests (Kells et al., 2001; Sousa et al., 2008; Lu et al., 2009; Hansen et al., 2012; Pandiselvam et al., 2017). O_3_ treatment caused 100% larval mortality of *E. cautella* after 24-h exposure at two temperature regimes (Husain et al., 2015). O_3_ at 2.0 ppm induced 83 and 27% mortality of *E. cautella* adults and larvae, respectively, after 12-h exposure (Abo-El-Saad et al., 2011). Three-day exposure to 5–45 ppm O_3_ led to 92–100% mortality of larvae of *Tribolium castaneum* Herbst, *S. zeamais* adults, and *P. interpunctella* in stored maize (Kells et al., 2001) and other stored products (Abo-El-Saad et al., 2011; Husain et al., 2015). *Tribolium confusum* du Val and *Ephestia kuehniella* Zeller showed different susceptibilities to O_3_ reflush treatment at 30-min intervals for 5 h at different developmental stages, and *T. confusum* was more tolerant than *E. kuehniella* at all developmental stages (Isikber and Oztekin, 2009). Together, these findings indicate that various MA combinations are available to create hypoxia and/or hypercapnia, and different MA combinations can be used for different pests in stored products.

## Changes in Energy/Nutrient Sources Under Modified Atmosphere

High-CO_2_ stress suppresses the production of NADPH and subsequently, glutathione, which are involved in the protection against the toxic effects of reactive oxygen species (Boardman et al., 2011). Further, NADPH contributes to nucleotide synthesis, cholesterol synthesis, and fatty-acid synthesis (Feron, 2009). Trehalose is the primary carbohydrate in insects, and plays an important role in insect development and all physiological activities by serving as an instant energy source as well as by mitigating abiotic stressors (Shukla et al., 2015). Trehalose protects cells against various environmental stresses, such as heat, cold, desiccation, dehydration, and oxidation. Chen and Haddad (2004) reported that trehalose can protect *Drosophila* and mammalian cells from hypoxic and anoxic injury. The mechanism underlying this protective action might be related to the decrease in protein denaturation through protein-trehalose interactions (Chen et al., 2003). In the presence of trehalose, cells can be maintained in the dry state for up to 5 days. Moreover, trehalose reportedly protects cultured human corneal epithelial cells from death by desiccation (Chen et al., 2003). Trehalose-6-phosphate synthase (TPS), which produces trehalose, is vital to insect growth and development (Chen et al., 2018). Overexpression of TPS increased trehalose levels and tolerance to anoxia (Chen et al., 2003). Trehalose plays an important role in protecting flies against anoxia injury, and induction of TPS increased tolerance to anoxia by reducing anoxia-induced protein aggregation (Tang et al., 2018).

Several studies have demonstrated that stored-product insect pests have the genetic potential to develop resistance to MA. In *Liposcelis bostrychophila* Badonnel, this resistance is related to enhanced levels of triacylglycerol and polysaccharides (Wang et al., 2000; Wang and Zhao, 2003). However, contents of energy substances, including polysaccharides, soluble proteins, and lipids, decreased in a dose- and time-dependent manner in response to CO_2_ in larvae of *S. paniceum* and *L. serricorne* (Cao et al., 2016a) and adult *S. paniceum* (Cao et al., 2016b) and *O. surinamensis* (Cao et al., 2015b). In bean weevil (*Callosobruchus chinensis* Linnaeus), Cui et al. (2017) reported that the levels of carbohydrates, amino acids, and organic acids increased, whereas those of free fatty acids decreased in response to hypoxia. When hypercapnia was added, these changes were further enhanced, except for the decrease in free fatty acids (Cui et al., 2017).

Hypoxia-adapted flies tend to have decreased glycolysis and pyruvate carboxylase fluxes relative to the amount of O_2_ consumed, and tend to produce acetate rather than oxaloacetate (Feala et al., 2009). In addition, in hypoxia-adapted flies, fewer protons are generated and more ATP per glucose is produced, pyruvate carboxylase flux is lower, and complex I rather than complex II was used in the electron transport chain. Based on simulations, it has been suggested that ATP-per-O_2_ efficiency is greater in hypoxia-adapted metabolism in insects (Harrison and Haddad, 2011). During metabolic processes, the production of cytochrome oxidase and mitochondrial ATP is significantly affected by O_2_ (Hochachka et al., 1996). Under very low atmospheric O_2_ partial pressure or temperature, ATP production is directly limited, which results in reduced rates of feeding, digestion, absorption, and protein synthesis (Harrison and Haddad, 2011). Under hypoxia, besides the direct effects on ATP levels, the AMP-to-ATP ratio increases because AMP kinases are activated upon AMP accumulation. Accordingly, multiple cellular effects related to the control of energy metabolism and growth have been observed (Tao et al., 2010). In *D. melanogaster* Meigen flies adapted to severe hypoxia, Feala et al. (2009) suggested a network-level hypothesis of metabolic regulation, in which lower baseline rates of biosynthesis resulted in lower anaplerotic flux and consequently, lower rates of glycolysis, less acidosis, and more efficient substrate use.

## Changes in Metabolic Enzymes in Response to Modified Atmosphere Gases

In insects, CO_2_ is thought to inhibit respiratory enzymes at concentrations higher than 20%; however, the effect varies strongly among species (Zhou et al., 2001). In *S. paniceum* and *Lasioderma serricorne* Fabricius, carboxyl esterase activity increased compared to that in the normal condition after exposure to a CO_2_-enriched atmosphere (Li et al., 2007, 2009). Acid phosphatase activity also increased under CO_2_ stress with the extension of exposure time, whereas alkaline phosphatase was hardly affected (Li et al., 2008). In *Araecerus fasciculatus* Degeer, the activities of carboxyl esterase and acid phosphatase increased significantly under CO_2_-enriched MA (75% CO_2_, 5% O_2_ and 20% N_2_) for 3 h (Li et al., 2012), and glutathione-S-transferase (GST) activity also increased significantly in *S. paniceum*, *L. serricorne*, and *A. fasciculatus* under the same condition (Li and Li, 2009). In *L. serricorne*, *LsGSTd1* (encoding GST) did not change significantly following exposure to CO_2_ stress, whereas the expression levels of *LsGSTt1* and *LsGSTs1* were significantly increased (Xu et al., 2017).

The expression of antioxidant enzymes, including catalase, superoxide dismutase (SOD), GST, and glutathione peroxidase (GPx), was reportedly increased in *Achaea janata* Linnaeus subjected to different oxidative stress stimuli, which also slowed down its development and resulted in weight reduction (Pavani et al., 2015). In pupae of *Anastrepha suspensa* Loew, the total antioxidant capacity was increased by more than twofold after 1 h of anoxic exposure (López-Martínez and Hahn, 2012). The increase was maintained for 24 h and was associated with increases in mitochondrial SOD (MnSOD) and GPx, but not catalase. Further, after 2-h anoxic exposure, cytoplasmic SOD (Cu-ZnSOD) activity was significantly increased when compared to normoxia (López-Martínez and Hahn, 2012).

## Molecular Mechanisms Underlying Adaption of Insects to Modified Atmospheres

Hypoxia is generally defined as <21% O_2_ and hyperoxia as >21% O_2_. In addition to gases such as CO_2_, O_3_, and NO, CA can be used as a pest control practice. Insights into the molecular mechanisms underlying the responses in insects to hypoxic/hypercapnic conditions are required to efficiently use MAs for pest control. Local hypoxia causes a rise in NO production in certain tissues of *Drosophila* larvae, and overexpression of NO synthase causes a greater hypoxia response, whereas knockout of protein kinase G or inhibition of NO synthase reduces such responses (Harrison and Haddad, 2011). Through microarray and bioinformatics analyses, Zhou et al. (2009) identified genes (*e.g.*, Notch pathway genes) that play important roles in the development of hypoxia resistance. Genes related to metabolism (*e.g*., carbon metabolism) were largely downregulated, whereas upregulated genes mainly encoded multiple components of epidermal growth factor (EGF), insulin, Notch, Toll, and immune deficiency (IMD) signal transduction (Zhou and Haddad, 2013). In addition, genes involved in protein digestion and tricarboxylic acid cycle as well as genes encoding stress-responsive heat shock proteins were increased in insects challenged by O_2_ deprivation (Cheng et al., 2012). Identification of the molecules that mediate the adaptation to hypoxia might lead to new therapeutic targets to protect or reverse hypoxia-induced pathologies (Zhou et al., 2009).

Under hypoxia, cells and tissues are challenged by O_2_ deprivation to the extent that energy production is inefficient. Trehalose reportedly protects *Drosophila* and mammalian cells from hypoxic and anoxic injury (Chen and Haddad, 2004). In mammalian cells transfected with the *Drosophila tps1* gene, the exogenous trehalose could protect the cells from hypoxic injury (Chen et al., 2003). Hypoxia-inducible factor, which is a key molecule produced in response to O_2_ deprivation, is mainly regulated by prolyl hydroxylase domain-containing enzymes (Hochachaka and Rupert, 2003; Wang et al., 2015). Organisms show different responses to constant hypoxia (CH) and intermittent hypoxia (IH), and the effect of hypoxia depends on the severity and duration of hypoxia (Farahani et al., 2008). In *D. melanogaster*, hypoxia resistance has been well studied. Severe short-term CH (2.5 h, 1% O_2_) and IH (cycles of 1–21% O_2_) triggered the expression of genes involved in immunity and unfolded protein, carboxylic acid, amino acid, and lipid metabolism (Azad et al., 2009; Zhou and Haddad, 2013). More importantly, gene families activated in response to CH include those involved in the metabolism of chitin, lipid, and carboxylic acid; the immune response; and the response to protein unfolding (Harrison and Haddad, 2011). Gene expression under CH and IH varies in both the number of responsive genes and the gene families affected. In a study by Zhou and Haddad (2013), gene families overrepresented in CH-treated flies included those involved in the response to unfolded proteins, lipids, carboxylic acid, amino acid metabolic processes, and immunity, whereas gene families overrepresented in IH were related to drug resistance. During CH exposure, strong upregulation of the chaperones heat shock protein *HSP70* and *HSP23* was observed in *D. melanogaster* (Harrison and Haddad, 2011). Overexpression of *HSP70*, which regulates CH tolerance, had no effect on IH tolerance, and overexpression of *Mdr49* enhanced adult survival under IH, but not CH (Zhou and Haddad, 2013). In *Sarcophaga crassipalpis* Macquart, *HSP* genes play a key role in the response to severe hypoxia (3% O_2_), with different HSPs having different functions (Michaud et al., 2011). Cryoprotective low-molecular-weight sugars and polyols can stabilize biological membranes and protect them from ice damage (Kostál et al., 2007; Overgaard et al., 2007), as do HPSs (*e.g*., *HSP70*) (Yi and Lee, 2003; Kostál and Tollarová-Borovanská, 2009).

Genes related to RNA editing are also involved in anoxia tolerance. For example, pre-mRNA adenosine deaminase plays an important role in IH tolerance through altering protein structure and function (Harrison and Haddad, 2011). Recent evidence suggests that atypical guanyl cyclases, which are heme-containing heterodimeric enzymes that are activated by hypoxia, but not NO (Morton, 2004) may mediate at least some of the rapid neuronal responses to O_2_ as conventional guanyl cyclases (Vermehren et al., 2006). NO-sensitive guanyl cyclases may also play a role in hypoxic responses (Wingrove and O’Farrell, 1999). Soluble guanylyl cyclases (sGCs) play a role in the synthesis of the intracellular messenger cyclic guanosine monophosphate (cGMP), and conventional sGCs are the main receptor for and mediate the majority of physiological actions of NO (Garthwaite, 2010). Atypical sGC subunits bind O_2_ to their heme group in a manner analogous to NO binding to the conventional sGCs under normal atmospheric conditions. Atypical sGCs have a relatively low affinity for O_2_, a property that is necessary for a molecular O_2_ detector that can sensitively detect a reduction in O_2_ concentration from the atmospheric level (Vermehren et al., 2006). The physiological effects of cGMP are typically mediated by activation of a cGMP-dependent protein kinase, a cyclic nucleotide-gated ion channel, or a cGMP-regulated phosphodiesterase (Lucas et al., 2000).

## Potential Role of Modified Atmospheres in Stored-Product and Fruit Pest Control

A study by Cui et al. (2017) showed that insect tolerance to hypoxia or hypoxia/hypercapnia is on the rise. The authors provided direct evidence of insect adaption to hypoxia, and reported free fatty acid regulation by hypercapnia in stored-product pests (Cui et al., 2017). Combined hypoxia exposure and low temperature or high CO_2_/NO has been used to sterilize commodities in postharvest pest management programs, and the current knowledge on the mechanisms involved in insect cross-tolerance can be used to develop more targeted control measures (Follett et al., 2018). However, one important problem is that many insects develop stronger resistance or cold cross-tolerance through physiological adaptions (Nilson et al., 2006; Cui et al., 2014). Therefore, more in-depth research is needed for the development and application of control measures in future.

At low temperature, MAs can increase pest mortality induced by low-temperature sterilization, and sometimes, the treatment duration can be shortened. Therefore, combined low temperature and hypoxia exposure have been used to sterilize commodities for pest control (Boardman et al., 2015; Saha et al., 2015). The control efficacy for storage pests can be enhanced by reducing O_2_ levels and increasing treatment time or temperature (Liu and Haynes, 2016). For example, Neven et al. (2014) indicated that heat treatment in combination with high CO_2_ and low O_2_ may be effective for the control of diapausing codling moth, *Cydia pomonella* Linnaeus, in walnut; especially, temperatures higher than 44°C rapidly killed the moths (Neven et al., 2014). Thus, high-temperature forced-air treatment combined with an O_2_-depleted and CO_2_-enriched atmosphere is an environmentally friendly postharvest mitigation approach to control quarantine pests (Johnson and Neven, 2010). In future, high or low temperature combined with low O_2_ and high CO_2_ or NO might have the potential to control or kill not only storage pests, but also fruit pests. Specialized machinery or technology for MA/temperature treatment can be developed for postharvest pest management (Villers et al., 2008; Mditshwa et al., 2018).

## Conclusion

MAs provide a highly effective non-chemical control measure for stored-product pests. The control effect of MAs can be reasonably improved through combination with temperature stress, or by using suitable facilities and techniques or other measures. Although some combination approaches (*e.g.*, combination with natural enemies) and related underlying mechanisms (*e.g.*, cross-tolerance of pests) remain to be resolved, MA control systems should be further developed, improved, and applied in stored-product protection for their unique advantages.

## Author Contributions

YC, WY, and CL conceived and designed manuscript structure. YC, KX, XZ, YB, and CL wrote the paper.

### Conflict of Interest Statement

The authors declare that the research was conducted in the absence of any commercial or financial relationships that could be construed as a potential conflict of interest.
